# Editorial: Transport and Membrane Traffic in Stomatal Biology

**DOI:** 10.3389/fpls.2022.898128

**Published:** 2022-05-31

**Authors:** Yizhou Wang, Rucha Karnik, Carlos Garcia-Mata, Honghong Hu

**Affiliations:** ^1^College of Agriculture and Biotechnology, Institute of Crop Science, Zhejiang University, Hangzhou, China; ^2^Laboratory of Plant Physiology and Biophysics, Plant Science Group, Institute of Molecular, Cell and Systems Biology, University of Glasgow, Glasgow, United Kingdom; ^3^Instituto de Investigaciones Biológicas, CONICET-Universidad Nacional de Mar del Plata, Mar del Plata, Argentina; ^4^College of Life Science and Technology, Huazhong Agricultural University, Wuhan, China

**Keywords:** stomata, guard cell, transport, membrane traffic, stomatal model

Global climate change and increasing atmospheric CO_2_ are clearly set to impact precipitation and the availability of fresh water worldwide. Agricultural water usage is fundamentally connected to plant gas exchange and hence to the carbon cycle of the globe, thereby affecting agricultural productivity (Blatt et al., 2017). The stomata of plants are tiny pores that facilitate the gas exchange between plants and the environment. Their movements are regulated by dynamic changes in the volume of guard cells that line the stomatal pore. Guard cell volume is regulated in response to environmental cues, balancing leaf gas exchange. Stomatal opening facilitates CO_2_ uptake for photosynthetic carbon assimilation and stomatal closing prevents water loss through transpiration (Lawson and Blatt, 2014). Although the total area of stomatal pores only represents <3% of the leaf surface, almost all CO_2_ absorption and up to 95% of water loss from plants are through these pores in stomata-bearing plant species (Schroeder et al., 2001). Indeed, changes in stomatal behavior in response to changing climatic conditions will, in turn, affect global water and carbon cycles (Lawson and Blatt 2014). Moreover, stomatal guard cells respond to an array of extracellular and intracellular signals, including light and CO_2_. In guard cells, ion transport and membrane vesicle traffic are coordinated for the regulation of stomatal aperture in response to environmental and endogenous cues (Sutter et al., 2007; Grefen et al., 2015; Jezek and Blatt, 2017; Xia et al., 2019). Thus, understanding stomatal function and their regulating mechanisms are vital for water use efficiency (WUE) in agriculture, and for the development of strategies for improved crops that are resilient to global climate changes.

In plants, water loss by transpiration occurs through the stomatal pore. WUE is calculated as a ratio of the amount of water used in metabolism to the water plant transpired and takes into account photosynthetic rate (A) to transpire rate (E), or the ratio of biomass produced by the plant to the rate of transpiration. However, improving WUE by enhancing plant A is difficult as enhancing CO_2_ uptake comes at a cost of water loss in most plants. Thereby, accelerating stomatal responses to reduce transpirational water loss is considered an efficient way to improve WUE. In the past decades, efforts are ongoing to improve WUE in crops, and success in such technologies requires fundamental knowledge of stomatal regulation in plants.

Quantitative modeling is a potential strategy to solve the dilemma of balancing between WUE and A. It offers a promising *in silico* approach to investigate the stomatal function and its related WUE. The OnGuard model is the first quantitative guard cell model with a set of macroscopic descriptors of guard-cell-specific features (Chen et al., 2012; Hills et al., 2012). Experimental tests (Chen et al., 2012; Hills et al., 2012; Wang et al., 2012, 2014; Minguet-Parramona et al., 2016; Jezek et al., 2021) established the reliability of the representations encoded in the model across a wide range of experimentation and led to a more profound understanding of the complex mechanisms behind many of the responses of guard cells and stomata to environmental change (Wang et al., 2017). OnGuard model offers users an unprecedented tool with which to explore the mechanics of stomatal functions across scales from the molecule to the whole plant. In this Research Topic, Shafaque et al. describe a standard protocol for using OnGuard Software in studying stomatal physiology and its related ion transport and homeostasis. They provided comprehensive guidance to the fresh users to manipulate the function of each key option of the model and took several examples to show how to change parameters in ion transporter characters and humidity.

Recent studies have shown that enhanced stomatal kinetics for opening and closure can contribute to high WUE in plants (Lawson and Blatt, 2014). Papanatsiou et al. (2019) expressed a synthetic, light-gated K^+^ channel, BLINK1 in the guard cells of Arabidopsis to accelerate both stomatal opening under light exposure and closing after irradiation, driving a 2.2-fold increase in biomass in fluctuating light without cost in water use by the plant. These findings open a new avenue for strategies toward improving plant WUE by using optogenetic tools that enhance stomatal kinetics. In this Research Topic, Ding and Chaumont provide insights into the regulation of stomatal kinetics by optimizing the expression of water transporting aquaporin proteins in plants.

In addition to the stomatal kinetics, stomatal morphology is also a key factor to affect stomatal response and WUE. The Poaceae family have distinctive dumbbell-shaped guard cells and specialized subsidiary cells to form an efficient stomatal complex. These allow for a faster grass stomatal response than any other stomatal type (Chen et al., 2017). However, the molecular mechanism of Poaceae family stomatal regulation is not well characterized. In this Research Topic, Wang and Chen highlight the unique structure and developmental progress of grass stomata and outline the different guard cell signaling mechanisms in monocots and eudicots.

In the last article of this Research Topic, Ren et al. provide us a mini-review on sulfur compounds in stomatal regulation. Sulfur is one of the essential elements for plants. It has important effects on plant growth, development, and abiotic and biotic responses. In recent studies, various sulfur compounds, including H_2_S, SO_2_, ${\text{SO}}_{3}^{2-}$, etc., were reported to regulate stomatal movements. This mini-review offers us the detail of how these sulfur compounds affect stomatal movements.

To sum up, this Research Topic provides up-to-date stomatal knowledge using multi-disciplinary approaches, including computational biology, agricultural biology, and molecular and cellular biology. We believe that stomatal research will continue to flourish in the future as novel technologies emerge.

## Author Contributions

All authors listed have made a substantial, direct, and intellectual contribution to the work and approved it for publication.

## Funding

This work was supported by the National Natural Science Foundation of China (31871537 and U2003115), Zhejiang Provincial Natural Science Foundation of China (LR21C020001), Hainan Yazhou Bay Seed Laboratory Science and Technology Program (B21HJ0220), the BBSRC (BB/S017348/1 and BB/S506734/1), the Royal Society London (RG160493, RF_ERE_210026, and UF150364), Wellcome (204820/Z/16/Z), and the University of Glasgow.

## Conflict of Interest

The authors declare that the research was conducted in the absence of any commercial or financial relationships that could be construed as a potential conflict of interest.

## Publisher's Note

All claims expressed in this article are solely those of the authors and do not necessarily represent those of their affiliated organizations, or those of the publisher, the editors and the reviewers. Any product that may be evaluated in this article, or claim that may be made by its manufacturer, is not guaranteed or endorsed by the publisher.

## References

[B1] Blatt M. R. Brodribb T. J. Torii K. U. (2017). Small pores with a big impact. Plant Physiol. 174, 467–469. 10.1104/pp.17.00642 28584063 PMC5462035

[B2] Chen Z. H. Chen G. Dai F. Wang Y. Hills A. Ruan Y. L.. (2017). Molecular evolution of grass stomata. Trends Plant Sci. 22, 124–139. 10.1016/j.tplants.2016.09.005 27776931

[B3] Chen Z. H. Hills A. Baetz U. Amtmann A. Lew V. L. Blatt M. R. (2012). Systems dynamic modeling of the stomatal guard cell predicts emergent behaviors in transport, signaling, and volume control. Plant Physiol. 159, 1235–1251. 10.1104/pp.112.197350 22635112 PMC3404696

[B4] Grefen C. Karnik R. Larson E. Lefoulon C. Wang Y. Waghmare S.. (2015). A vesicle-trafficking protein commandeers Kv channel voltage sensors for voltage-dependent secretion. Nature Plants 1, 15108. 10.1038/nplants.2015.166 27250541

[B5] Hills A. Chen Z. H. Amtmann A. Blatt M. R. Lew V. L. (2012). OnGuard, a computational platform for quantitative kinetic modeling of guard cell physiology. Plant Physiol. 159, 1026–1042. 10.1104/pp.112.197244 22635116 PMC3387691

[B6] Jezek M. Blatt M. R. (2017). The membrane transport system of the guard cell and its integration for stomatal dynamics. Plant Physiol. 174:487–519. 10.1104/pp.16.01949 28408539 PMC5462021

[B7] Jezek M. Silva-Alvim F. Hills A. Donald N. Ishka M. R. Shadbolt J.. (2021). Guard cell endomembrane Ca^2+^-ATPases underpin a ‘carbon memory' of photosynthetic assimilation that impacts on water-use efficiency. Nat. Plants. 7:1301–1313. 10.1038/s41477-021-00966-2 34326530

[B8] Lawson T. Blatt M. R. (2014). Stomatal size, speed, and responsiveness impact on photosynthesis and water use efficiency. Plant Physiol. 164, 1556–1570. 10.1104/pp.114.237107 24578506 PMC3982722

[B9] Minguet-Parramona C. Wang Y. Hills A. Vialet-Chabrand S. Griffiths H. Rogers S.. (2016). An optimal frequency in Ca^2+^ oscillations for stomatal closure is an emergent property of ion transport in guard cells. Plant Physiol. 170, 33–42. 10.1104/pp.15.01607 26628748 PMC4704601

[B10] Papanatsiou M. Petersen J. Henderson L. Wang Y. Christie J. M. Blatt M. R. (2019). Optogenetic manipulation of stomatal kinetics improves carbon assimilation, water use, and growth. Science. 363:1456–1459. 10.1126/science.aaw0046 30923223

[B11] Schroeder J. I. Allen G. J. Hugouvieux V. Kwak J. M. Waner D. (2001). Guard cell signal transduction. Annu. Rev. Plant Physiol. Plant Mol. Biol. 52, 627–658. 10.1146/annurev.arplant.52.1.627 11337411

[B12] Sutter J. Sieben C. Hartel A. Eisenach C. Thiel G. Blatt M. R. (2007). Abscisic acid triggers the endocytosis of the Arabidopsis KAT1 K^+^ channel and its recycling to the plasma membrane. Curr. Biol. 17, 1396–1402. 10.1016/j.cub.2007.07.020 17683934

[B13] Wang Y. Hills A. Blatt M. R. (2014). Systems analysis of guard cell membrane transport for enhanced stomatal dynamics and water use efficiency. Plant Physiol. 164, 1593–1599. 10.1104/pp.113.233403 24596330 PMC3982726

[B14] Wang Y. Hills A. Vialet-Chabrand S. Papanatsiou M. Griffiths H. Rogers S.. (2017). Unexpected connections between humidity and ion transport discovered using a model to bridge guard cell-to-leaf scales. Plant Cell 29, 2921–2939. 10.1105/tpc.17.00694 29093213 PMC5728137

[B15] Wang Y. Papanatsiou M. Eisenach C. Karnik R. Williams M. Hills A.. (2012). Systems dynamic modeling of a guard cell CL- channel mutant uncovers an emergent homeostatic network regulating stomatal transpiration. Plant Physiol. 160, 1956–1967. 10.1104/pp.112.207704 23090586 PMC3510123

[B16] Xia L. Marques-Bueno M. Bruce C. Karnik R. (2019). Unusual roles of secretory SNARE SYP132 in plasma membrane H^+^-ATPase traffic and vegetative plant growth. Plant Physiol. 180, 837–858. 10.1104/pp.19.00266 30926657 PMC6548232

